# First person – Claire Simon

**DOI:** 10.1242/bio.040105

**Published:** 2018-12-15

## Abstract

First Person is a series of interviews with the first authors of a selection of papers published in Biology Open, helping early-career researchers promote themselves alongside their papers. Claire Simon is first author on ‘A Gata4 nuclear GFP transcriptional reporter to study endoderm and cardiac development in the mouse’, published in BIO. Claire is a postdoctoral fellow in the lab of Anna-Katerina Hadjantonakis at the Sloan Kettering Institute, Memorial Sloan Kettering Cancer Center, USA, investigating cell fates and dynamic signaling pathways.


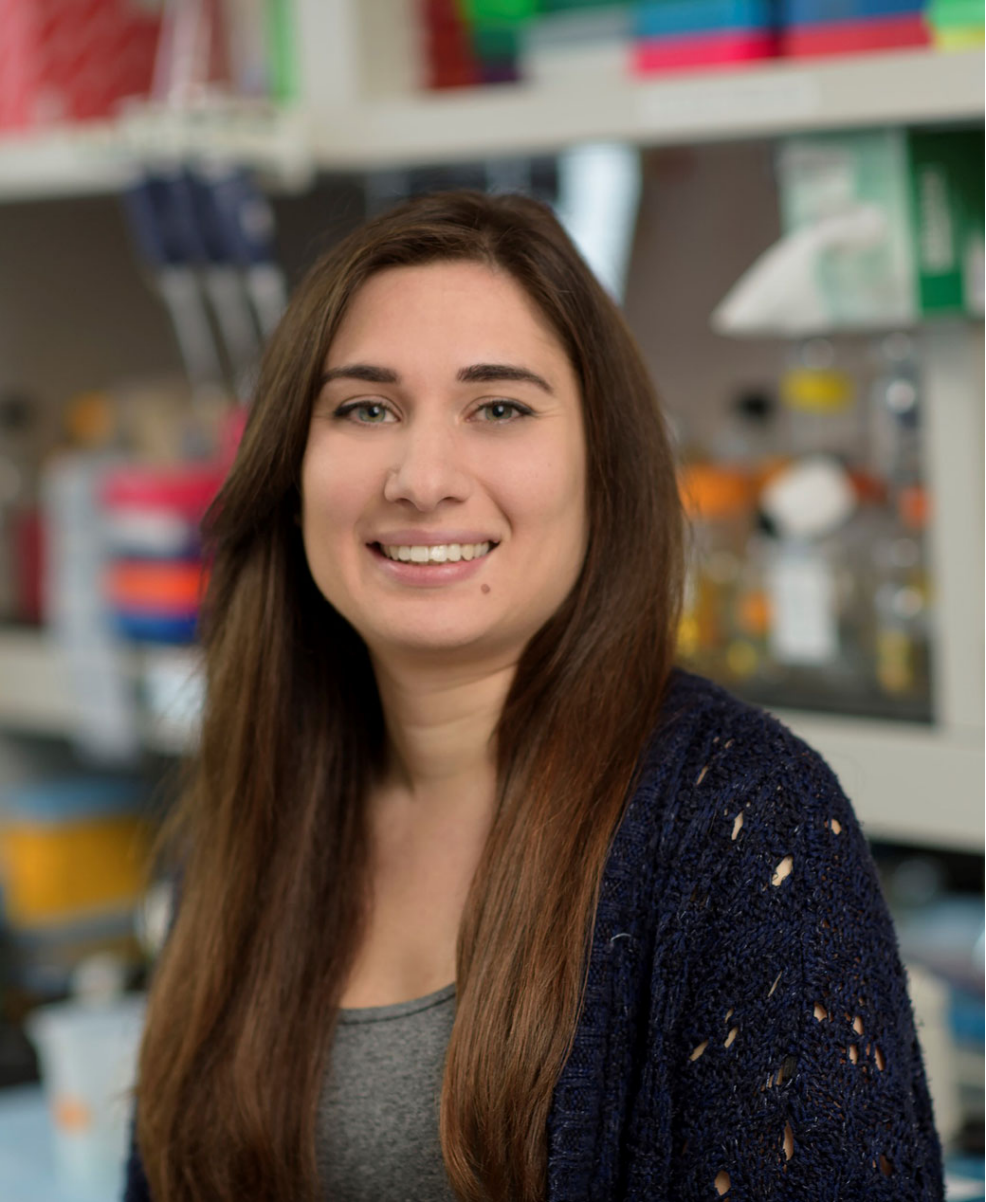


**Claire Simon**

**What is your scientific background and the general focus of your lab?**

I am a developmental biologist. During my undergraduate degree I became fascinated with how cell fates are specified and patterned during embryogenesis. This led me to pursue a developmental biology PhD under the supervision of Liz Robertson at the University of Oxford, UK. My project focused on the transcriptional regulation of cell fate specification during mouse gastrulation – a critical time when the embryonic body plan is established and the pluripotent embryonic epiblast is specified to the primary germ layers; ectoderm, mesoderm and endoderm. After my PhD I joined Kat Hadjantonakis' lab at the Memorial Sloan Kettering Cancer Center, NY, USA as a postdoctoral fellow. The lab is interested in cell fate specification and morphogenesis during mouse development, with expertise on the development and use of quantitative imaging tools.

**How would you explain the main findings of your paper to non-scientific family and friends?**

Embryonic cells initially have the potential to become any cell of the adult organism. During development, embryonic cells become gradually more and more specialized to generate the diverse range of cell types found in the body. When cells specialize, genes associated with particular cell types are switched on. For example, *Gata4* is a gene that is switched on in heart (cardiac) and gut (endoderm) cells. By genomically engineering the *Gata4* gene, we were able to generate a mouse that turns on a green fluorescent protein (GFP) when *Gata4* is on. This enables us to follow *Gata4* expressing heart and gut cells in the embryo, revealing when and where these specialized cell types emerge during development.

**What are the potential implications of these results for your field of research?**

Gata4 plays important roles in the differentiation of multiple tissue cell types including heart, ovary, testes, pancreas, lung, liver and the extra-embryonic endoderm. The mouse line we have generated is a nuclear localized *Gata4* transcriptional reporter, which accurately reflects endogenous gene expression and is ideal for time-lapse imaging and single-cell resolution analysis. This mouse line can also be used to generate a tagged form of the GATA4 protein to perform biochemical assays. We anticipate that this mouse line will provide a useful tool for others in the field to study *Gata4* expression, transcriptional regulation and protein interactions in a variety of biological contexts.

**What has surprised you the most while conducting your research?**

What surprised me in my research is that lineage-specific genes, rather than being master regulators and/or markers for just one particular cell fate, are often co-opted in multiple and potentially very diverse cell types and function in complex context-dependent ways. *Gata4* is a perfect example of a versatile transcription factor, with different roles in numerous cell types of mesoderm and endoderm origin (both embryonic and extra-embryonic) and throughout development from pre-implantation into adulthood.

**What, in your opinion, are some of the greatest achievements in your field and how has this influenced your research?**

The field of developmental biology has greatly advanced due to new technologies in imaging, for example light sheet microscopy, the improvement in fluorescent proteins for live-imaging and the ease of generating reporters with CRISPR-Cas9 gene targeting. Together, these have provided insights into developmental processes with unprecedented resolution. One challenge is how to quantitatively analyze and interpret these kinds of data. During my post-doc I've been fortunate to benefit from expertise within our lab, from collaborators and from our institution's courses to develop the computational and statistical skills needed for quantitative image analysis, which has been invaluable for my research.

**Figure:**
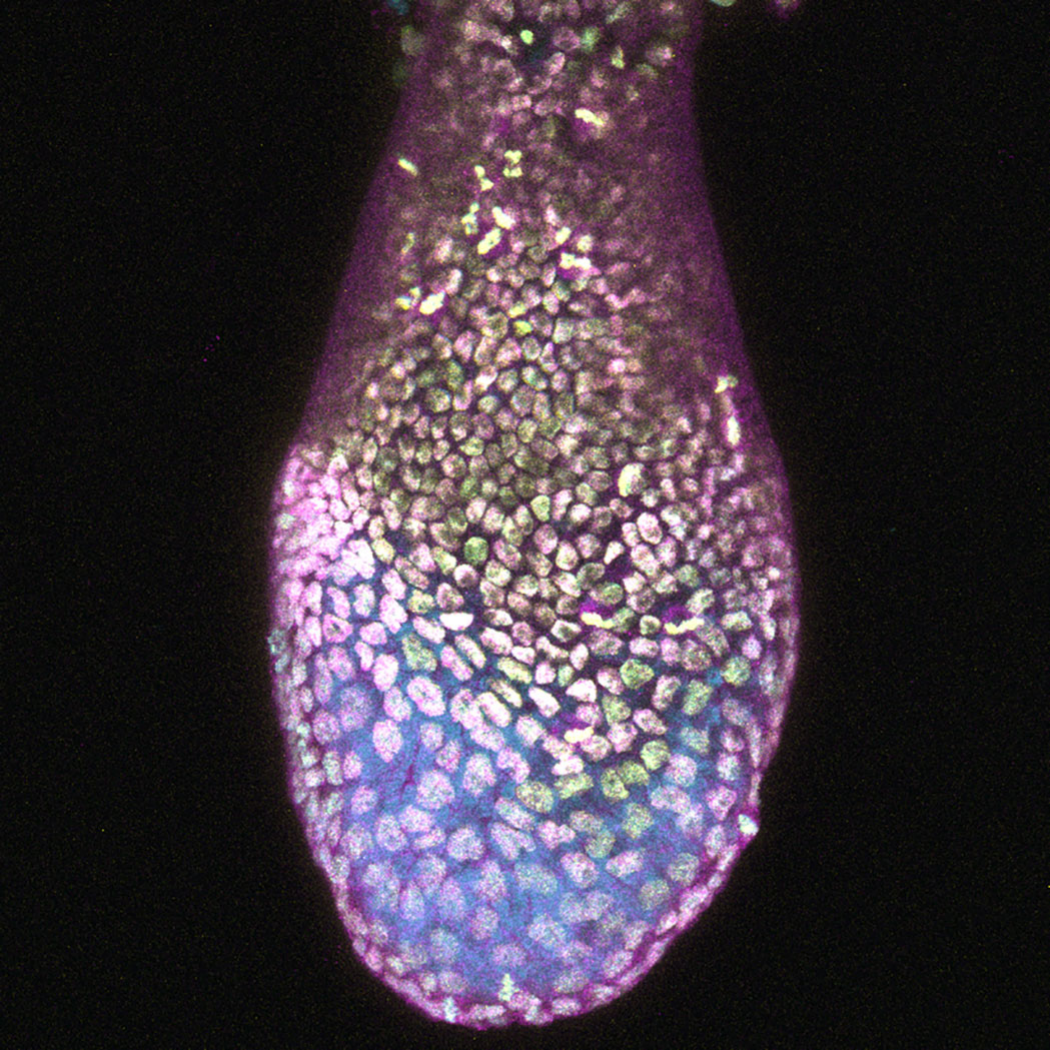
**The GATA zinc-finger transcription factor GATA4 is important for endoderm and cardiac development.** We generated a novel nuclear localized fluorescent reporter to study Gata4 expression in the mouse. Shown here is a projection of a confocal z-stack of an embryonic day 6.5 (E6.5) Gata4H2B-GFP/+ mouse embryo expressing the Gata4 transcriptional reporter (yellow), immunostained for GATA4 (magenta) and counterstained with Hoechst (cyan) to detect nuclei. Gata4H2B-GFP is expressed in the outer layer of the embryo, the visceral endoder.

**What changes do you think could improve the professional lives of early-career scientists?**

The current system for post-doctoral research doesn't leave much room for a work/life balance. The pressure to publish, perusing scarce funding opportunities, short term contracts and low pay leave post-docs feeling over-worked and under-valued. Aside from institutions providing more financial support and job security, there needs to be a shift in attitudes and expectations within academia to allow early-career scientists the freedom to find their work/life balance; this would improve mental health, prevent burnout and ultimately increase happiness and productivity.

**What's next for you?**

I received a post-doctoral fellowship to continue my work in the Hadjantonakis lab researching the establishment of pluripotency *in vivo*. My project will focus on understanding the molecular mechanisms driving cell fate specification within the inner cell mass of the pre-implantation mouse embryo to either the pluripotent epiblast or the extra-embryonic primitive endoderm. I am particularly interested in the dynamics of FGF4 signaling in initiating this cell fate decision, and the downstream effectors mediating lineage-specific outputs of FGF/ERK signaling.
